# Cervical cancer mortality: descriptive study and time series analysis, Mato Grosso, 2013-2022

**DOI:** 10.1590/S2237-96222025v34e20240503.en

**Published:** 2025-08-08

**Authors:** Gabriela Omena Silva, Yara de Souza Braga, Izabela de Figueiredo Reis, Luiz Eduardo Alessio, Vanessa de Almeida Raia, Alexandra Secreti Prevedello, Aline Morandi Alessio

**Affiliations:** 1 Universidade Federal de Mato Grosso, Faculdade de Medicina, Sinop, MT, Brazil; 2 Faculdade Garça Branca Pantanal, Programa de Pós-Graduação em Ortodontia, Cuiabá, MT, Brazil

**Keywords:** Cervical Neoplasms, Epidemiology, Mortality, Women’s Health, Time Series Studies, Neoplasias del Cuello Uterino, Epidemiología, Mortalidad, Salud de la Mujer, Estudios de Series Temporales

## Abstract

**Objective:**

To analyze the temporal trend and sociodemographic profile of cervical cancer mortality in the state of Mato Grosso between 2013-2022.

**Methods:**

Time series study, with data from the Mato Grosso Health Department. The age-adjusted mortality rate variation was calculated using Joinpoint software and sociodemographic variables were analyzed using the Chi-square test, through the SPSS Statistics.

**Results:**

There were 1,071 deaths, with annual percentage change and mean annual percentage variation of -0.81 (95% confidence interval [95%CI -3.30; 1.81] p-value 0.516). The highest number of deaths occurred among women over 64 years of age (32.8%), with incomplete basic education (62.0%), light black skin (62.8%), single (32.3%), working in paid jobs (52.5%), residing in the Central-West/Northwest macro-region (53.9%), who received medical assistance (75.7%) and underwent medical tests (36.8%).

**Conclusion:**

In Mato Grosso, cervical cancer mortality presented a stationary trend in the time series. Deaths predominated among women over 64 years old and residing in the Central-West/Northwest macro-region.

## Resumen

**Objetivo:**

Analizar la tendencia temporal y el perfil sociodemográfico de la mortalidad por cáncer de cuello uterino en Mato Grosso entre 2013-2022.

**Métodos:**

Estudio de series temporales, con datos del Departamento de Salud de Mato Grosso. La variación de la tasa de mortalidad ajustada por edad se calculó mediante el software Joinpoint y las variables sociodemográficas se analizaron mediante la prueba de Chi-cuadrado, utilizando el programa SPSS Statistics.

**Resultados:**

Se registraron 1.071 fallecimientos, con un cambio porcentual anual y una media de cambio porcentual anual de -0,81 (intervalo de confianza del 95% [IC95%] -3,30; 1,81; p-valor 0,516). El mayor número de muertes ocurrió entre mujeres mayores de 64 años (32,8%), con educación primaria incompleta (62%), pardas (62,8%), solteras (32,3%), remuneradas (52,5%), residentes en la macrorregión Centro-Norte/Noroeste (53,9%), que recibieron asistencia médica (75,7%) e investigación (36,8%).

**Conclusión:**

En Mato Grosso, la mortalidad por cáncer de cuello uterino mostró una tendencia estacionaria en la serie temporal. Las defunciones predominaron entre mujeres mayores de 64 años y residentes de la macrorregión Centro-Norte/Noroeste.

**Ethical aspects**

This research used public domain data and anonymized databases.

## Introduction

Cervical cancer, excluding non-melanoma skin cancer, is the fourth most common malignant neoplasm and the fourth leading cause of cancer deaths in women worldwide (1). In Brazil, it ranks third among the most diagnosed types of cancer and has the fourth highest mortality rate in the female population. The Central-West region has the third highest mortality rate, exceeding the country’s average rates, with Mato Grosso recording an incidence rate higher than the Central-West average and a percentage of screening performed lower than regional and national rates (2).

Approximately 95.0% of cervical cancers originate from persistent infection with oncogenic serotypes of Human Papillomavirus (HPV), mainly sexually transmitted HPV-16 and HPV-18. It is estimated that more than 80.0% of the sexually active population will become infected with HPV. In 75.0% to 90.0% of cases, the infection is transient, regressing within two years, without the need for intervention (3). The interval between acquisition of infection and progression to invasive carcinoma can be 15 to 20 years or more, providing a window of opportunity for screening and treatment of premalignant lesions (3,4).

Mortality from cervical cancer is distributed heterogeneously, being higher in developing countries and those marked by socioeconomic inequalities. In developed countries, there was a reduction in incidence and mortality due to the effective implementation of screening programs (5).

In 2020, the World Health Organization launched Global strategy to accelerate the elimination of cervical cancer as a public health problem which set three targets for 2030: 90.0% coverage of HPV vaccination for girls at age 15; screening coverage (70.0% of women undergoing high-performance testing between ages 35-45); 90.0% treatment of pre-cancerous lesions and invasive cancer (6).

Vaccination against HPV is a strategy for primary prevention of cervical cancer. Currently, following international recommendations, the vaccination schedule comprises a single dose, maintaining the level of protection and aiming to increase vaccination coverage (7). Immunization seeks to combat the initial HPV infection and is highly effective among young women who were seronegative (7,8).

In Brazil, screening is performed using a cervical oncotic cytology test, provided by the Unified Health System free of charge for women who have started their sexual life from the age of 25 to 64, promoting access to early diagnosis (9). The HPV DNA test for virus detection has greater sensitivity compared to cytology, optimizing screening time and costs (10).

The aim of this study was to analyze the temporal trend and sociodemographic profile of cervical cancer mortality in the state of Mato Grosso between 2013-2022. It is believed that deaths are influenced by sociodemographic factors. It is expected, as is the case in Brazil, that the mortality rate in Mato Grosso will be declining, due to the screening offered by the Unified Health System. This study aims to contribute to the development of more targeted and effective strategies, with an impact on reducing mortality, considering the scarcity of epidemiological studies on the subject.

## Methods

This is an epidemiological, descriptive, and historical trend study.

The state of Mato Grosso, located in the Central-West region, has an area of 903,208.36 km^2^ and an estimated population of 3,658,649 inhabitants, of which 49.7% (1,817,408) are women (11).

It has 141 municipalities, divided into 16 health regions, grouped into six health macro-regions. In 2021, the Central North macroregion had the largest population, with 1,028,372 inhabitants, followed by the North with 794,433, South with 543,133, Central Northwest with 531,559, East with 348,769 and West with 320,968 (12).

Data on deaths was extracted from the Data Warehouse platform from the Information Systems repository of the State Department of Health of Mato Grosso, available on the website http://appweb3.saude.mt.gov.br/dw. The platform collects and organizes information transferred by municipalities and subsequently sends it to the Mortality Information System of the Unified Health System’s Information Technology Department.

Deaths of female residents of Mato Grosso due to cervical cancer, registered in the Information System of the Mato Grosso State Health Department between 2013-2022 were analyzed.

Deaths obtained considered the 10th Revision of the International Classification of Diseases (ICD-10). Malignant neoplasm of the cervix corresponds to code C53. According to the proportional redistribution methodology published by the World Health Organization, deaths due to malignant neoplasm of the uterus, unspecified portion (ICD-10, C55) were redistributed equally between deaths due to cervical cancer and deaths due to endometrial cancer (13). The 50.0% of deaths classified in ICD-10, C55 attributed to cervical cancer were added to deaths due to malignant neoplasm of the cervix (ICD-10, C53) by correction and used in the study.

To describe the sociodemographic characteristics of deaths, variables that reflect sociodemographic aspects were selected, aiming to outline a profile of cervical cancer mortality in the state and identify possible social disparities that may influence these indicators.

The variables collected were year of death, age group, race/skin color, marital status, occupation, education, macro-region, municipality of residence, medical care, tests performed and outpatient, home, or hospital testing.

The occupation variable was reclassified to group activities that imply direct financial compensation as paid occupation, highlighting retirees, pensioners, teachers, and domestic workers. Activities that do not result in direct financial compensation, such as stay-at-home spouses, students and the unemployed, were categorized as unpaid occupations. The reclassification was carried out considering the considerable number of registered occupations, enabling the identification of relevant patterns among the different occupational groups.

As of 2020, the Mato Grosso State Health Department reclassified some municipalities, creating the new Center- Northwest macro-region, which included part of the municipalities of the former Center-North. For this study, these regions were unified as Center-North/Northwest (Table 1).

**Table 1:** Distribution of municipalities according to the macro-region of Mato Grosso

Macro-region	Municipality
Central-North/Northwest	Acorizal, Alto Paraguai, Arenápolis, Aripuanã, Barão de Melgaço, Barra do Bugres, Brasnorte, Campo Novo do Parecis, Castanheira, Chapada dos Guimarães, Colniza, Cuiabá, Denise, Diamantino, Jangada, Juína, Juruena, Nobres, Nortelândia, Nossa Senhora do Livramento, Nova Brasilândia, Nova Marilândia, Nova Maringá, Nova Olímpia, Poconé, Porto Estrela, Rosário Oeste, Santo Antônio do Leverger, São José do Rio Claro, Sapezal, Santo Afonso, Tabaporã, Tangará da Serra, Várzea Grande.
East	Água Boa, Araguaiana, Barra do Garças, Bom Jesus do Araguaia, Campinápolis, Canarana, Cocalinho, Confresa, General Carneiro, Nova Nazaré, Nova Xavantina, Novo São Joaquim, Pontal do Araguaia, Ponte Branca, Porto Alegre do Norte, Querência, Ribeirão Cascalheira, Santa Cruz do Xingu, Santa Terezinha, São Félix do Araguaia, São José do Xingu, Torixoréu, Vila Rica.
North	Alta Floresta, Carlinda, Cláudia, Colíder, Feliz Natal, Guarantã do Norte, Itanhangá, Itaúba, Juara, Lucas do Rio Verde, Marcelândia, Matupá, Nova Bandeirantes, Nova Canaã do Norte, Nova Monte Verde, Nova Mutum, Nova Ubiratã, Paranaíta, Peixoto de Azevedo, Santa Carmem, Sinop, Sorriso, Tapurah, Terra Nova do Norte, União do Sul, Vera.
West	Araputanga, Cáceres, Campos de Júlio, Comodoro, Glória d’Oeste, Jauru, Mirassol d’Oeste, Nova Lacerda, Pontes e Lacerda, Porto Esperidião, Reserva do Cabaçal, Rio Branco, Rondolândia, Salto do Céu, São José dos Quatro Marcos, Vale de São Domingos, Vila Bela da Santíssima Trindade.
South	Alto Araguaia, Alto Garças, Alto Taquari, Araguaína, Campo Verde, Dom Aquino, Guiratinga, Itiquira, Jaciara, Juscimeira, Paranatinga, Pedra Preta, Poxoréo, Primavera do Leste, Rondonópolis, Santo Antônio do Leste, São Pedro da Cipa, Tesouro.

To control classification bias, the occupation and health region categories were standardized and the proportional redistribution of deaths due to unspecified malignant neoplasm of the uterus (C55) was applied, following the methodology of the World Health Organization (13).

Data exported from the Data Warehouse platform were organized into spreadsheets in Microsoft Excel 2016. Descriptive analysis of variables and chi-square test of adherence were performed using the SPSS Statistics software, version 25.0.

To verify the distribution of deaths according to the municipality of residence, a map was created in TerraView, version 5.6.5. The import of geospatial data and the estimate of the female population in Mato Grosso, distributed by year, was obtained from the Brazilian Institute of Geography and Statistics.

The mortality rate was calculated per 100,000 women. The variation in the mortality rate was assessed using the Joinpoint Trend Analysis software, version 5.3.0, considered an appropriate method for time series analysis (14). The Joinpoint regression model was used to calculate age-adjusted mortality rates, using a logarithmic approach


​Log(Yt)=β
0+β
1⋅
X

where: log Yt (mortality rate in the year); β0 (intercept); β1 (mortality variation rate over time) and X (year of death).

Based on β1 and the standard error, the Annual Percentage Variation (APV) rates were calculated:


APV=(−1+10β
1)×
100

The Mean Annual Percentage Variation (AAPV) was estimated as the weighted average of the AAPVs. The 95% confidence intervals (95%CI) are: 


IC95% minimum=(10β
1minimum−1)×
100

IC95% maximum=(10β
1maximum−1)×
100

The temporal trend of the rates was interpreted based on the APV and p-value (significance level of 5.0%), being: stationary (p-value>0.05), increasing (positive APV and p-value<0.05) or decreasing (negative APV and p-value<0.05).

The article was prepared in accordance with the Reporting of Observational Studies in Epidemiology (STROBE) guidelines.

## Results

**Figure 1:**
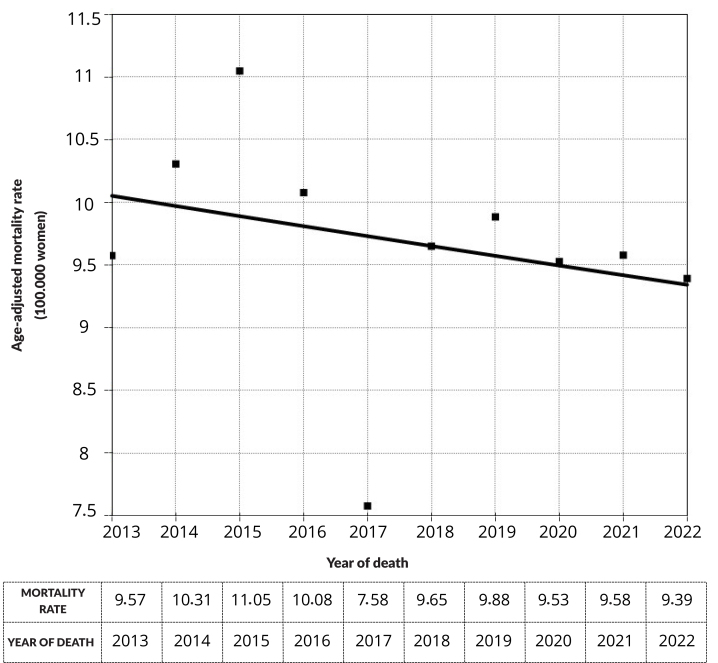
Variation in age-adjusted mortality rate from cervical cancer. Mato Grosso, 2013-2022

Between 2013-2022, there were 1,071 deaths from cervical cancer in Mato Grosso. The age-adjusted mortality rate had an Annual Percentage Variation (APV) and Mean Annual Percentage Variation (AAPV) -0.81; [95%CI -3.30; 1.81; p-value 0.516)], with a stationary temporal trend throughout the period (Figure 1).

Table 2 describes the sociodemographic profile of deaths, considering: ICD-10 classification, age group, race/skin color, marital status, education, occupation, and macro-regions of residence. ICD-10, C53, malignant neoplasm of the cervix, presents the highest number of deaths, with 964 cases (90.0%; p-value<0.001).

**Table 2:** Characterization of deaths from cervical cancer. Mato Grosso, 2013-2022 (n=1,071)

Variables	n (%)	p-value
**International Classification of Diseases**		
53	964 (90.0)	<0.001
55	107 (10.0)	
**Age group** (years)		
<25	2 (0.2)	<0.001
25-29	27 (2.5)	
30-39	125 (11.7)	
40-49	230 (21.5)	
50-59	227 (21.2)	
60-64	108 (10.1)	
>64	352 (32.8)	
**Race/skin color**		
Brown	673 (62.8)	<0.001
White	291 (27.2)	
Black	81 (7.5)	
Indigenous	14 (1.3)	
Yellow (Asian)	6 0.6)	
Ignored/Blank	6 (0.6)	
**Marital status**		
Single	346 (32.3)	<0.001
Married	292 (27.3)	
Consensual conjugal union	72 (6.7)	
Legally separated/divorced	86 (8.0)	
Widower	217 (20.3)	
Ignored/Blank	58 (5.4)	
**Education** (years)		
0	188 (17.6)	<0.001
1-3	174 (16.2)	
4-7	302 (28.2)	
8-11	263 (24.6)	
>12	63 (5.8)	
Ignored	81 (7.6)	
**Occupation**		
Paid	562 (52.5)	<0.001
Unpaid	467 (43.6)	
Blank/ignored	42 (3.9)	
Macro-region		
Center-North/Northwest	577 (53.9)	<0.001
South	177 (16.5)	
North	154 (14.4)	
West	86 (8.0)	
East	76 (7.1)	
Blank/ignored	1 (0.1)	

Deaths were mainly among those over 64 years of age (352 deaths/32.8%), followed by the age group between 40-49 years (230 deaths/21.5%) (p-value<0.001); single women (346 deaths/32.3%) (p-value<0.001); with education between 4 and 7 years of study (302 deaths/28.2%) (p-value<0.001); and light black population with (673 deaths/62.8%) (p-value<0.001). Regarding occupation, 562 deaths (52.5%) occurred in women who had a paid occupation, and 467 deaths (43.6%) were among women with unpaid occupations (p-value<0.001). 

**Figure 2:**
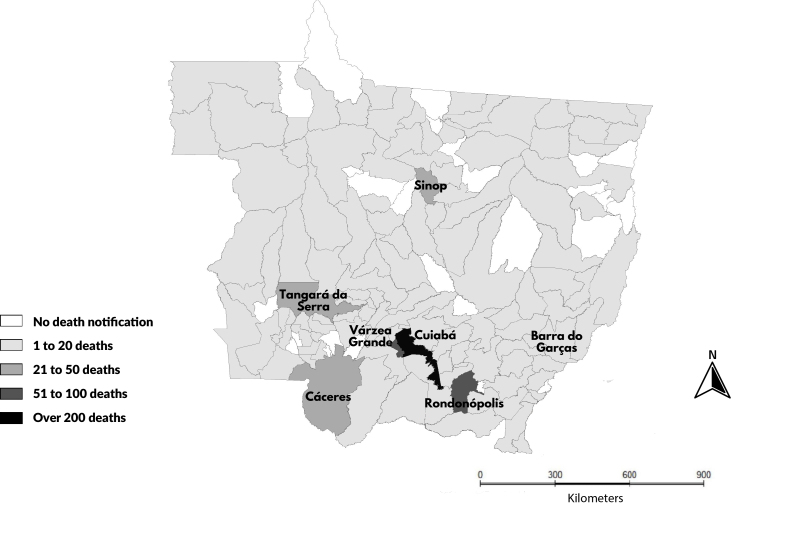
Geospatial distribution of deaths from cervical cancer according to place of residence. Mato Grosso, 2013-2022

The highest mortality by macroregion occurred in the Center-North/Northwest (577 deaths; 53.9%), followed by the South (177 deaths; 16.5%), North (154 deaths; 14.4%); West (86 deaths; 8.0%), and East (76 deaths; 7.1%) (p-value<0.001), with the following cities being respectively those with most deaths: Cuiabá (26.2%), Rondonópolis (8.0%); Sinop (2.9%), Cáceres (4.0%); Barra do Garças (1.6%) (Figure 2).

Most of the deaths, 811 (75.7%), occurred in patients who received medical care (p-value<0.001). However, only 394 deaths (36.8%) were investigated (p-value 0.008). It should be noted that these women had limited access to outpatient, hospital, and home testing (p-value<0.001) (Table 3).

**Table 3:** Deaths from cervical cancer, according to the variables: medical care, tests done, outpatient, hospital, and home tests. Mato Grosso, 2013-2022 (n=1,071)

Variables	n (%)	p-value
**Medical assistance**		
Yes	811 (75.7)	<0.001
No	24 (2.2)	
Ignored/blank	236 (22.1)	
**Research carried out**		
Yes	394 (36.8)	0.008
No	312 (29.1)	
Ignored/blank	365 (34.1)	
**Outpatient tests**		
Yes	49 (4.6)	<0.001
No	1022 (95.4)	
**Home tests**		
Yes	102 (9.5)	<0.001
No	969 (90.5)	
**Hospital tests**		
Yes	173 (16.2)	<0.001
No	898 (83.8)	

## Discussion

The study analyzed the temporal trend and sociodemographic profile of cervical cancer mortality in Mato Grosso between 2013-2022. The variation trend in the mortality rate was stationary. There were more deaths among women over 64 years old, with incomplete primary education, mixed race, single, with a paid occupation, living in the Central-North/Northwest macro-region, who received medical assistance and underwent medical testing.

In Brazil, cervical cancer screening is still performed using colpocytological examination. In 2023, Brazil started a Pilot Project for the Control and Elimination of Cervical Cancer, using the HPV-DNA test for screening (15). The test has been considered the gold standard by the World Health Organization since 2014, as it is more effective and sensitive than colpocytology, has a lower cost, fewer false-negative results and is part of global strategies to eliminate the disease (16). It can be performed from the age of 30, replacing colpocytology, which is done only when the HPV-DNA test is positive and, if negative, repeated every 5 years (17). 

The benefits in reducing the incidence and mortality from cervical cancer generated by screening with HPV detection tests outweigh the harm caused by the increase in positive tests and colposcopies. They allow screening to begin at an older age with more spaced testing intervals (18) and can also be performed by self-collection. Self-collection is easy to perform, with less discomfort and can overcome geographical barriers (19).

This study identified a stationary temporal trend, over the period in the mortality rate in Mato Grosso between 2013-2022. However, a time series study carried out in Brazil in 2023 demonstrated a statistically significant decrease in cervical cancer mortality rates in the country in 2020, compared to the previous decade, in women over 40 years of age (20). This stagnation may indicate limitations in screening programs and the need for more effective strategies to expand adherence and access in the state.

Few deaths were observed in women under the age of 25, justifying the exclusion of this population from screening. Women over 64 years of age, although not included in the screening, represent the most prominent age group, indicating a possible deficiency in screening in the previous decade. Delaying the diagnosis of these women can increase mortality. A 2023 analytical observational study, carried out in São Paulo, analyzed 207 abnormal cervical smear results in women over 64 years of age and observed that those who did not follow screening guidelines had a greater chance of developing cancer and precursor lesions, suggesting a review of the screening age range in Brazil (21). These findings highlight the need for active reaching out by health agents and campaigns to promote cytology at an earlier age, aiming to reduce mortality and treatment costs.

A cross-sectional study in Paraná, in 2013, evaluated reasons why women did not adhere to the exam. Delay in care, distance to the health unit, shame, embarrassment, and discomfort at the time of the examination were the factors reported. These feelings may be due to constant sexual repression, especially among older women, resulting in the neglect of their gynecological demands and creating a barrier to knowledge about the importance of prevention (22). Carrying out campaigns to demystify the exam, promoting a welcoming environment and training professionals for a humanized collection with minimal discomfort for the patient are actions that could contribute to increasing screening engagement.

A scoping review carried out in Brazil in 2022 analyzed different educational interventions for the prevention of cervical cancer and reinforced that group discussions, lectures and information leaflets can increase women’s knowledge and improve their adherence to screening. It also showed a significant increase in screening rates in interventions led by health workers (23). Focusing on awareness campaigns for prevention through cytology collection and vaccination for HPV and active reaching out to women over 50 years of age by health agents can be a strategy to improve screening rates in the state.

It is worth noting that women over 40 years of age did not have access to HPV vaccination coverage, which has been made available by the Unified Health System since 2014 for girls aged 9-14. Mortality rates are expected to fall in the coming years, as the impact of vaccination in reducing HPV infection has been shown to be significant worldwide (24).

Data from the Ministry of Health from December 2024 shows that Brazil is increasing the immunized population, with coverage of 96.6% for girls aged 14 and 69.0% between 9 and 14 years old (25). In Mato Grosso state, vaccination coverage was considered adequate (above 80.0%) only regarding the first dose, with 58.8% for the second dose, especially among 16-year-old girls (26). Single-dose vaccination, starting in 2024, will make it easier to achieve the state’s vaccination coverage target and increase vaccination uptake (7), especially among groups most likely to miss the second dose.

In the Brazilian Institute of Geography and Statistics Census (2022), 58.0% of the population of Mato Grosso declared themselves to be brown (light black skin), which explains that most of the deaths were among brown women. A 2020 Brazilian observational ecological study, analyzing cervical cancer mortality between 1996-2017, highlighted mortality in the brown population in the North and Northeast regions and Black population in the South and Southeast. In the Central-West region, the Indigenous population stood out, diverging from the present study. The population’s skin color is related to an increased risk of mortality, considering possible socioeconomic and cultural factors, low screening coverage, inadequate treatments, and advanced stage at diagnosis (27).

The study also highlighted the higher prevalence of mortality among single women. The hypotheses for this data are that marital status can influence quality of life, health, and survival rate. The lower mortality among married women is possibly related to greater social and psychological support, in addition to generally adopting healthier lifestyle habits (27). Single women also tend to have greater exposure to HPV infection due to the possibility of having more sexual partners (20,28).

The highest number of deaths occurred in women with incomplete primary education. Similar data was found in a retrospective descriptive study carried out in Cascavel-Paraná, which analyzed 90 deaths between 2012-2021, of which 31.1% occurred in the 4 to 7 years of schooling group (29). These data are reinforced by a systematic review carried out in Brazil in 2018, which analyzed the relationship between education and cervical cancer, highlighting that educational barriers can make it difficult for women to understand adherence, prevention, and the disease progression process (30).

Among the deaths involving people in unpaid jobs, a higher proportion was observed among stay-at-home spouses and, to a lesser extent, students and the unemployed. An observational study in Recife, state of Pernambuco, in 2008, analyzed 323 deaths and demonstrated even higher proportions, with 69.0% of deaths among stay-at-home spouses (31). In line with the findings, another descriptive cross-sectional study, carried out in the Northeast, analyzed 699 deaths between 2013-2014 and found that women who work outside the home have greater participation in screening (32).

The results indicate low disease investigation and deficiency in preventive tests. In Mato Grosso, 6.3% of women aged 25-64 have never undergone a cytopathological test. In the Central-West region, the rate of those tested was 78.8%, below the national average of 81.3% (2). However, most of the women who died received medical assistance, indicating the presence of an oncology care network in the state.

 A descriptive study in Brazil analyzed mortality from cervical cancer between 2012-2016, recording 27,716 deaths. The highest rates occurred in the North and Central-West, where the rate fell from 27.12 to 24.13 per 100,000 women (33). These data reinforce the high mortality rate from cervical cancer in the region and indicate challenges in early detection and access to health services.

The sparse distribution in the Central-West makes access to health services difficult, requiring strategies for effective and equitable coverage (34,35). In Mato Grosso, a descriptive study showed that coverage of the Family Health Strategy in the 141 municipalities remained below 70.0%. The Baixada Cuiabana area, including Cuiabá and Várzea Grande, recorded the lowest rates at approximately 40.0%, highlighting a shortage in the primary network (36).

A 2020 systematic review identified barriers to cervical cancer screening in rural areas, such as distance, lack of transportation, and access to specialists (37). Mato Grosso, due to its territorial extension, faces challenges related to the distance between smaller municipalities and the main reference centers, a problem that could be mitigated with the use of self-testing for HPV.

The high death rate in some municipalities can be attributed to the centralization of medical centers and specialized oncology services in the state capital (36). Smaller municipalities may face underreporting or lack of access to adequate treatments, leading to patient migration to areas with medical care (38). This study showed a higher number of deaths in the Central-North/Northwest macro-region, especially in Cuiabá. A 2023 observational study confirmed this trend, highlighting São Paulo with the highest screening coverage (20).

As limitations of this study, it is noted that the research was carried out with secondary data, which may lead to selection bias due to underreporting of deaths or lack of diagnosis. Reporting cervical cancer is not mandatory. The Mato Grosso Health Department platform only allows investigation variables to be categorized as “yes” or “no,” with no options to detail or exclude data as “unknown” or “not informed,” which could compromise the accuracy of the results. Furthermore, the 50.0% cutoff point for redistributing deaths due to unspecified malignant neoplasm of the uterus (ICD-10, C55) is a valid practice, disseminated by the World Health Organization (13) and adopted by the Brazilian National Cancer Institute (39), but it can be limiting. It assumes an equal distribution between cervical and endometrial cancer and may not reflect the epidemiological reality. Only patients residing in Mato Grosso were selected, disregarding those who may have moved to other states for treatment.

To estimate the relative risk of an event such as death, it is necessary to compare two distinct groups: one that experienced the event (death) and one that did not (survivors). However, the data used in this study were extracted from a system that only records deaths. Therefore, there is no comparison group (survivors), making it impossible to calculate the relative risk of death. This method requires the proportion of occurrence of the event in two distinct groups to estimate the difference in risk between them and does not apply to the present study.

This is the first study to analyze the temporal trend and sociodemographic profile of cervical cancer mortality in Mato Grosso between 2013-2022. The results demonstrated a stationary trend in mortality, despite the downward trend observed in Brazil. This discrepancy suggests that screening and preventive policies may be less effective for some populations. As expected, sociodemographic characteristics, such as age group, race/skin color and macro-region of residence, describe the profile of deaths, highlighting inequalities in access and adherence to health programs.

To overcome these challenges, strategies such as awareness raising, expansion of vaccination, introduction of HPV molecular testing, professional training, and active reaching out for women over 50 for screening are suggested. Such measures could improve early detection, expand screening, reduce mortality, and optimize resources. 

The results of this study are expected to support the fight against cervical cancer in Mato Grosso. Understanding the sociodemographic characteristics of deaths can guide targeted health policies, reducing regional inequalities and promoting more effective prevention. Future studies should assess adherence and impact of current screening policies, aiming to adapt interventions to the specific needs of the state.

## Data Availability

The database and analysis variables used in this research are available at http://appweb3.saude.mt.gov.br/dw.

## References

[1] International Agency for Research on Cancer (IARC) (2022). Cancer TODAY. Data visualization tools for exploring the global cancer burden in 2022.

[2] Instituto Nacional de Câncer José Alencar Gomes da Silva (2023). Dados e números sobre câncer de colo do útero.

[3] Kusakabe M, Taguchi A, Sone K, Mori M, Osuga Y (2023). Carcinogenesis and management of human papillomavirus-associated cervical cancer. Int J Clin Oncol.

[4] Kombe AJ, Zoa-Assoumou S, Bounda GA, Nsole-Biteghe FA, Jin TC, Zouré AA (2023). Avanços no papel etiopatológico e controle do HPV na oncogênese do câncer cervical. Front Biosci.

[5] Souza GRM de, Cardoso AM, Pícoli RP, Mattos IE (2022). Perfil do rastreamento do câncer do colo do útero em Campo Grande, Mato Grosso do Sul: um estudo avaliativo do período 2006-2018. Epidemiol Serv Saúde.

[6] World Health Organization (2020). Global strategy to accelerate the elimination of cervical cancer as a public health problem.

[7] Ministério da Saúde (BR) (2024). Nota Técnica nº 41/2024-CGICI/DPNI/SVSA/MS: Atualização das recomendações da vacinação contra HPV no Brasil.

[8] Villa LL, Richtmann R (2023). HPV vaccination programs in LMIC: is it time to optimize schedules and recommendations?. J Pediatr.

[9] Carvalho CF, Teixeira JC, Bragança JF, Derchain S, Zeferino LC, Vale DB (2022). Cervical Cancer Screening with HPV Testing: Updates on the Recommendation. Rev Bras Ginecol Obstet.

[10] Instituto Nacional de Câncer José Alencar Gomes da Silva (2016). Diretrizes brasileiras para o rastreamento do câncer do colo do útero.

[11] Instituto Brasileiro de Geografia e Estatística (IBGE) (2022). Panorama - Censo Demográfico 2022.

[12] Brasil (2022). Núcleo de Gestão Estratégico para Resultados.

[13] Mathers CD, Bernard C, Iburg KM, Inoue M, Ma Fat D, Shibuya K (2003). Global Burden of Disease in 2002: data sources, methods and results.

[14] National Cancer Institute (NCI) (2024). Joinpoint regression program.

[15] Ministério da Saúde (BR) (2023). Portaria GM/MS nº 299, de 22 de março de 2023. Institui estratégia de mudança tecnológica para controle e eliminação do câncer do colo do útero, no âmbito da Política Nacional de Prevenção e Controle do Câncer, dentro do Sistema Único de Saúde - SUS.

[16] Koliopoulos G, Nyaga VN, Santesso N, Bryant A, Martin-Hirsch PP, Mustafa RA (2017). Cytology versus HPV testing for cervical cancer screening in the general population. Cochrane Database Syst Rev.

[17] Teixeira JC, Vale DB, Discacciati MG, Campos CS, Bragança JF, Zeferino LC (2023). Cervical cancer screening with DNA-HPV testing and precancerous lesions detection: a Brazilian population-based demonstration study. Rev Bras Ginecol Obstet.

[18] Ministério da Saúde (BR) (2024). Testagem molecular para detecção de HPV e rastreamento do câncer do colo do útero. Relatório de Recomendação nº 878.

[19] Souza CA de, Sena AB (2022). Identification of cervical self-collection as a cervical cancer screening tool. RSD.

[20] Luizaga CTM, Jardim BC, Wünsch V, Eluf J, Silva GAE (2023). Recent changes in trends of mortality from cervical cancer in Southeastern Brazil. Rev Saude Publica.

[21] Zago RA, Camilo-Júnior DJ, Ávilla SCGP, Xavier-Júnior JCC (2023). Underestimated cervical cancer among women over 65 years old: is it time to revise the screening target age group?. Rev Bras Ginecol Obstet.

[22] Silva MAS, Teixiera EMB, Ferrari RAP, Cestari MEW, Cardelli AAM (2015). Fatores relacionados a não adesão à realização do exame de Papanicolau. Rev Rene.

[23] Mariño JM, Nunes LMP, Ali YCMM, Tonhi L do C, Salvetti M de G (2023). Educational interventions for cervical cancer prevention: a scoping review. Rev Bras Enferm.

[24] Kamolratanakul S, Pitisuttithum P (2021). Human papillomavirus vaccine efficacy and effectiveness against cancer. Vaccines (Basel).

[25] Ministério da Saúde (BR) (2025). Vacinação HPV.

[26] Moura L de L, Codeço CT, Luz PM (2021). Cobertura da vacina papilomavírus humano (HPV) no Brasil: heterogeneidade espacial e entre coortes etárias. Rev bras epidemiol.

[27] Dantas DB, da Luz Costa T, da Silva ASA, de Campos Gomes F, de Melo-Neto JS (2020). Mortality from cervical cancer in Brazil: an ecological epidemiologic study of a 22-year analysis. Ecancermedicalscience.

[28] Costa TML, Heráclio S, Amorim MMR, Souza PRE, Lubambo N, Souza GF de A (2019). Human papillomavirus and risk factors for cervical adenocarcinoma in the state of Pernambuco, Brazil. Rev Bras Saude Mater Infant.

[29] Costa TML, Heráclio S, Amorim MMR, Souza PRE, Lubambo N, Souza GF de A (2019). Human papillomavirus and risk factors for cervical adenocarcinoma in the state of Pernambuco, Brazil. Rev Bras Saude Mater Infant.

[30] Dellabeta dos Santos C, Marin AF, Bernegozzi Bessa B, Bernegozzi Bessa V, de Araujo Sodré LK (2023). Aspectos epidemiológicos de mortalidade por câncer de colo do útero em Cascavel-PR durante o período de 2012 a 2021. Braz J Implantol Health Sci.

[31] Almeida KIV (2018). Desigualdade social e câncer do colo do útero: uma revisão sistemática.

[32] Mendonça VG de, Lorenzato FRB, Mendonça JG de, Menezes TC de, Guimarães MJB (2008). Mortalidade por câncer do colo do útero: características sociodemográficas das mulheres residentes na cidade de Recife, Pernambuco. Rev Bras Ginecol Obstet.

[33] Ribeiro JF, Silva ARV, Campelo VC, Santos SLD, Coêlho DMM (2015). Perfil sociodemográfico e clínico de mulheres com câncer do colo do útero em uma cidade do Nordeste. Rev G&S.

[34] Tallon B, Monteiro D, Soares L, Rodrigues N, Morgado F (2020). Tendências da mortalidade por câncer de colo no Brasil em 5 anos (2012-2016). Saúde Debate.

[35] Moreira DP, Santos MA da C, Pilecco FB, Dumont-Pena É, Reis IA, Cherchiglia ML (2022). Tratamento ambulatorial do câncer do colo do útero em tempo oportuno: a influência da região de residência de mulheres no Estado de Minas Gerais, Brasil. Cad Saude Publica.

[36] Barbosa IR, Souza DLB de, Bernal MM, Costa I do CC (2016). Desigualdades regionais na mortalidade por câncer de colo de útero no Brasil: tendências e projeções até o ano 2030. Ciênc Saude Coletiva.

[37] Martinelli NL, Scatena JHG, Castro M de L, Soares NRF, Charbel SC, Souza NF da S (2023). Análise da estruturação da Rede de Atenção à Saúde no estado de Mato Grosso, Brasil, no contexto da Regionalização. Ciênc Saude Coletiva.

[38] Atere-Roberts J, Smith JL, Hall IJ (2020). Interventions to increase breast and cervical cancer screening uptake among rural women: a scoping review. Cancer Causes Control.

[39] Galvão SM, Atanaka M, Sousa NF da S, Galvão ND (2022). Potential years of life lost to cancer in Mato Grosso, stratified by sex: 2000 to 2019. Rev Bras Epidemiol.

[40] Instituto Nacional de Câncer José Alencar Gomes da Silva (2022). Estimativa: 2023 Incidência de Câncer no Brasil.

