# Supplementary material for: Cervical cancer mortality: descriptive study and time series analysis, Mato Grosso, 2013-2022
Source: Epidemiol Serv Saude. 2025 Aug 8;34:e20240503. doi: 10.1590/S2237-96222025v34e20240503.en (PMC12342724; doi:10.1590/S2237-96222025v34e20240503.en)
Supplement: Translation

# Translation: Mortalidade por câncer de colo do útero: estudo descritivo e análise de série temporal, Mato Grosso, 2013-2022

**DOI:** 10.1590/S2237-96222025v34e20240503.pt

**Authors:** Gabriela Omena Silva, Yara de Souza Braga, Izabela de Figueiredo Reis, Luiz Eduardo Alessio, Vanessa de Almeida Raia, Alexandra Secreti Prevedello, Aline Morandi Alessio

**Affiliations:** 1 Universidade Federal de Mato Grosso, Faculdade de Medicina, Sinop, MT, Brasil; 2 Faculdade Garça Branca Pantanal, Programa de Pós-Graduação em Ortodontia, Cuiabá, MT, Brasil

**Keywords:** Neoplasias do Colo do Útero, Epidemiologia, Mortalidade, Saúde da Mulher, Estudos de Séries Temporais

## Abstract

**Objetivo:**

Analisar a tendência temporal e o perfil sociodemográfico da mortalidade por câncer de colo do útero no Mato Grosso entre 2013-2022.

**Métodos:**

Estudo de série temporal, com dados da Secretaria de Saúde de Mato Grosso. A variação da taxa de mortalidade ajustada por idade foi calculada pelo software Joinpoint e as variáveis sociodemográficas foram analisadas pelo teste Qui-quadrado, através do SPSS Statistics.

**Resultados:**

Ocorreram 1.071 óbitos, com variação percentual anual e variação percentual anual média de -0,81 (intervalo de confiança de 95% [IC95% -3,30; 1,81] p-valor 0,516). O maior número de óbitos ocorreu entre mulheres acima de 64 anos (32,8%), com ensino fundamental incompleto (62,0%), pardas (62,8%), solteiras (32,3%), remuneradas (52,5%), residentes na macrorregião Centro-Norte/Noroeste (53,9%), que receberam assistência médica (75,7%) e investigação (36,8%).

**Conclusão:**

Em Mato Grosso, a mortalidade por câncer de colo de útero apresentou tendência estacionária da série temporal. Os óbitos predominaram entre mulheres com mais de 64 anos e residentes na macrorregião Centro-Norte/Noroeste.

**Aspectos éticos**

Esta pesquisa utilizou bancos de dados de domínio público e anonimizados.:

## Introdução

O câncer de colo do útero, excluindo o câncer de pele não melanoma, é a quarta neoplasia maligna mais comum e a quarta maior causa de óbitos por câncer no mundo, em mulheres (1). No Brasil, ocupa o terceiro lugar entre os tipos de câncer mais diagnosticados e quarta maior taxa de mortalidade na população feminina. A região Centro-Oeste apresenta a terceira maior taxa de mortalidade, superando as taxas médias do país, com o Mato Grosso registrando taxa de incidência superior à média do Centro Oeste e percentual de realização de rastreamento inferior aos índices regionais e nacionais (2).

Aproximadamente 95,0% dos cânceres cervicais se originam a partir da infecção persistente por sorotipos oncogênicos do Papilomavírus Humano (HPV), principalmente o HPV-16 e HPV-18, transmitidos sexualmente. Estima-se que mais de 80,0% da população sexualmente ativa irá se infectar pelo HPV. Em 75,0% a 90,0% dos casos, a infecção é transitória, regredindo em até dois anos, sem necessidade de intervenção (3). O intervalo entre a aquisição da infecção até progressão para um carcinoma invasivo pode levar de 15 a 20 anos ou mais, oferecendo uma janela de oportunidade para o rastreamento e tratamento de lesões pré-malignas (3,4).

A mortalidade pelo câncer de colo do útero se distribui de maneira heterogênea, sendo mais elevada em países em desenvolvimento e marcados por desigualdades socioeconômicas. Nos países desenvolvidos, houve redução na incidência e mortalidade devido à implantação efetiva de programas de rastreamento (5).

Em 2020, a Organização Mundial de Saúde lançou *Global strategy to accelerate the elimination of cervical cancer as a public health problem* que estabeleceu três metas para 2030: 90,0% de cobertura da vacinação contra HPV para meninas aos 15 anos; cobertura de rastreamento (70,0% das mulheres submetidas a testes de alto desempenho entre 35-45 anos); 90,0% de tratamento de lesões pré-cancerosas e câncer invasivo (6).

A vacinação contra o HPV é estratégia para prevenção primária do câncer de colo uterino. Atualmente, seguindo as recomendações internacionais, o esquema vacinal é realizado com dose única, mantendo nível de proteção e visando aumento da cobertura vacinal (7). A imunização busca combater a infecção inicial por HPV, sendo altamente eficaz entre mulheres jovens que eram soronegativas (7,8).

No Brasil, o rastreio é realizado pelo exame de colpocitologia oncótica cervical, disponibilizado pelo Sistema Único de Saúde gratuitamente para mulheres que iniciaram vida sexual a partir de 25 anos até os 64 anos, promovendo acesso ao diagnóstico precoce (9). O teste de DNA-HPV para detecção do vírus apresenta maior sensibilidade em comparação com a citologia, otimiza o tempo e os custos do rastreamento (10).

O objetivo deste estudo foi analisar a tendência temporal e o perfil sociodemográfico da mortalidade por câncer de colo do útero no Mato Grosso entre 2013-2022. Acredita-se que os óbitos são influenciados por fatores sociodemográficos. Espera-se, assim como ocorre no Brasil, a taxa de mortalidade no Mato Grosso esteja em declínio, devido ao rastreamento oferecido pelo Sistema Único de Saúde. Este estudo visa contribuir para o desenvolvimento de estratégias mais direcionadas e eficazes, com impacto na redução da mortalidade, considerando a escassez de estudos epidemiológicos sobre o tema.

## Métodos

Trata-se de um estudo epidemiológico, descritivo e de tendência histórica.

O estado de Mato Grosso, localizado na região Centro-Oeste, possui uma área de 903.208,36 km^2^ e população estimada de 3.658.649 habitantes, sendo 49,7% (1.817.408) mulheres (11).

Possui 141 municípios, dividido em 16 regiões de saúde, agrupadas em seis macrorregiões de saúde. Em 2021, a macrorregião Centro Norte tinha a maior população, com 1.028.372 habitantes, seguida pela Norte com 794.433, Sul com 543.133, Centro Noroeste com 531.559, Leste com 348.769 e Oeste com 320.968 (12).

Os dados de óbitos foram extraídos da plataforma Data Warehouse do repositório de Sistemas de Informação da Secretaria de Estado de Saúde de Mato Grosso, disponível no site http://appweb3.saude.mt.gov.br/dw. A plataforma coleta, organiza informações transferidas pelos municípios e envia posteriormente para o Sistema de Informações sobre Mortalidade do Departamento de Informática do Sistema Único de Saúde.

Analisou-se os óbitos de mulheres residentes em Mato Grosso por câncer de colo de útero, registrados no Sistema de Informação da Secretaria de Estado de Saúde de Mato Grosso, entre 2013-2022.

Os óbitos foram obtidos considerando a 10ª Revisão da Classificação Internacional de Doenças (CID-10). A neoplasia maligna do colo do útero corresponde ao código C53. Conforme a metodologia de redistribuição proporcional, difundida pela Organização Mundial de Saúde, óbitos por neoplasia maligna do útero porção não especificada (CID-10, C55) foram redistribuídos igualmente entre óbitos por câncer de colo do útero e óbitos por câncer do corpo do útero (13). Os 50,0% dos óbitos classificados no CID-10, C55 atribuídos ao câncer de colo do útero foram somados aos óbitos por neoplasia maligna do colo do útero (CID-10, C53) pela correção e utilizados no estudo.

Para descrever as características sociodemográficas dos óbitos, selecionaram-se variáveis que refletem aspectos sociodemográficos, objetivando-se traçar um perfil da mortalidade por câncer de colo uterino no estado e identificar possíveis disparidades sociais que possam influenciar esses indicadores.

As variáveis coletadas foram: ano do óbito, faixa etária, raça/cor da pele, estado civil, ocupação, escolaridade, macrorregião, município de residência, assistência médica, investigação realizada e investigação ambulatorial, domiciliar ou hospitalar.

A variável ocupação foi reclassificada para agrupar as atividades que implicam em compensação financeira direta como ocupação remunerada, destacando-se aposentadas, pensionistas, professoras e empregadas domésticas. Atividades que o exercício não resulta em compensação financeira direta como donas de casa, estudantes e desempregadas foram categorizadas em ocupação não remunerada. A reclassificação foi realizada considerando o grande número de ocupações registradas, possibilitando identificação de padrões relevantes entre os diferentes grupos ocupacionais.

A partir de 2020, a Secretaria de Estado de Saúde de Mato Grosso reclassificou alguns municípios, criando a nova macrorregião Centro Noroeste, que incluiu parte dos municípios da antiga Centro Norte. Para este estudo, essas regiões foram unificadas como Centro Norte/Noroeste (Tabela 1).

**Tabela 1:** Distribuição dos municípios de acordo com a macrorregião do Mato Grosso

Macrorregião	Município
Centro-Norte/Noroeste	Acorizal, Alto Paraguai, Arenápolis, Aripuanã, Barão de Melgaço, Barra do Bugres, Brasnorte, Campo Novo do Parecis, Castanheira, Chapada dos Guimarães, Colniza, Cuiabá, Denise, Diamantino, Jangada, Juína, Juruena, Nobres, Nortelândia, Nossa Senhora do Livramento, Nova Brasilândia, Nova Marilândia, Nova Maringá, Nova Olímpia, Poconé, Porto Estrela, Rosário Oeste, Santo Antônio do Leverger, São José do Rio Claro, Sapezal, Santo Afonso, Tabaporã, Tangará da Serra, Várzea Grande.
Leste	Água Boa, Araguaiana, Barra do Garças, Bom Jesus do Araguaia, Campinápolis, Canarana, Cocalinho, Confresa, General Carneiro, Nova Nazaré, Nova Xavantina, Novo São Joaquim, Pontal do Araguaia, Ponte Branca, Porto Alegre do Norte, Querência, Ribeirão Cascalheira, Santa Cruz do Xingu, Santa Terezinha, São Félix do Araguaia, São José do Xingu, Torixoréu, Vila Rica.
Norte	Alta Floresta, Carlinda, Cláudia, Colíder, Feliz Natal, Guarantã do Norte, Itanhangá, Itaúba, Juara, Lucas do Rio Verde, Marcelândia, Matupá, Nova Bandeirantes, Nova Canaã do Norte, Nova Monte Verde, Nova Mutum, Nova Ubiratã, Paranaíta, Peixoto de Azevedo, Santa Carmem, Sinop, Sorriso, Tapurah, Terra Nova do Norte, União do Sul, Vera.
Oeste	Araputanga, Cáceres, Campos de Júlio, Comodoro, Glória d’Oeste, Jauru, Mirassol d’Oeste, Nova Lacerda, Pontes e Lacerda, Porto Esperidião, Reserva do Cabaçal, Rio Branco, Rondolândia, Salto do Céu, São José dos Quatro Marcos, Vale de São Domingos, Vila Bela da Santíssima Trindade.
Sul	Alto Araguaia, Alto Garças, Alto Taquari, Araguaína, Campo Verde, Dom Aquino, Guiratinga, Itiquira, Jaciara, Juscimeira, Paranatinga, Pedra Preta, Poxoréo, Primavera do Leste, Rondonópolis, Santo Antônio do Leste, São Pedro da Cipa, Tesouro.

Para o controle do viés de classificação, padronizou-se as categorias de ocupação e de região de saúde e aplicou-se a redistribuição proporcional dos óbitos por neoplasia maligna do útero porção não especificada (C55), seguindo a metodologia da Organização Mundial de Saúde (13).

Os dados exportados da plataforma Data Warehouse foram organizados em planilhas no Microsoft Excel 2016. Análise descritiva das variáveis e teste qui-quadrado de aderência foram feitos no programa SPSS Statistics, versão 25.0.

Para verificar a distribuição dos óbitos conforme o município de residência elaborou-se um mapa no TerraView, versão 5.6.5. A importação dos dados geoespaciais e estimativa da população feminina, distribuída por ano, no Mato Grosso foi obtida pelo Instituto Brasileiro de Geografia e Estatística.

A taxa de mortalidade foi calculada a cada 100 mil mulheres. A variação da taxa de mortalidade foi avaliada utilizando o Joinpoint Trend Analysis Software, versão 5.3.0, considerado método adequado para análise de séries temporais (14). Empregou-se o modelo de regressão do Joinpoint para calcular as taxas de mortalidade ajustadas por idade, empregando uma abordagem logarítmica:


​Log(Yt)=β
0+β
1⋅
X

em que: log Yt (taxa de mortalidade no ano); β0 (intercepto); β1 (taxa de variação da mortalidade ao longo do tempo) e X (ano do óbito).

Com base em β1 e no erro-padrão, calcularam-se as taxas de Variação Percentual Anual (VPA):


VPA=(−1+10β
1)×
100

Estimou-se a Variação Percentual Anual Média (VPAM) como a média ponderada das VPAs. Os Intervalos de Confiança de 95% (IC95%) são: 


IC95% mínimo=(10β
1mínimo−1)×
100

IC95% máximo=(10β
1máximo−1)×
100

A tendência temporal das taxas foi interpretada com base na VPA e p-valor (nível de significância de 5,0%), sendo: estacionária (p-valor>0,05), crescente (VPA positiva e p-valor<0,05) ou decrescente (VPA negativa e p-valor<0,05).

Elaborou-se o artigo em conformidade com as diretrizes do *Reporting of Observational Studies in Epidemiology* (STROBE).

## Resultados

Entre 2013-2022, ocorreram 1.071 registros de óbitos por câncer de colo do útero no Mato Grosso. A taxa de mortalidade ajustada por idade teve Variação Percentual Anual (VPA) e Variação Percentual Anual Média (VPAM) -0,81; IC95% -3,30; 1,81; p-valor 0,516)], com tendência temporal estacionária ao longo do período (Figura 1).

**Figura 1:** Variação da taxa de mortalidade ajustada por idade do câncer de colo do útero. Mato Grosso, 2013-2022

A Tabela 2 apresenta a descrição do perfil sociodemográfico dos óbitos, considerando: CID-10, faixa etária, raça/cor da pele, estado civil, escolaridade, ocupação e macrorregiões de residência. O CID-10, C53, referente à neoplasia maligna de colo do útero, apresenta o maior número de óbitos com 964 casos (90,0%; p-valor<0,001).

**Tabela 2:** Caracterização dos óbitos por câncer de colo de útero. Mato Grosso, 2013-2022 (n=1.071)

Variáveis	n (%)	p-valor
**Classificação Internacional de Doenças**		
53	964 (90,0)	<0,001
55	107 (10,0)	
**Faixa etária** (anos)		
<25	2 (0,2)	<0,001
25-29	27 (2,5)	
30-39	125 (11,7)	
40-49	230 (21,5)	
50-59	227 (21,2)	
60-64	108 (10,1)	
>64	352 (32,8)	
**Raça/cor da pele**		
Parda	673 (62,8)	<0,001
Branca	291 (27,2)	
Preta	81 (7,5)	
Indígena	14 (1,3)	
Amarela	6 (0,6)	
Ignorado/em branco	6 (0,6)	
**Estado civil**		
Solteiro	346 (32,3)	<0,001
Casado	292 (27,3)	
União consensual	72 (6,7)	
Separado judicialmente/divorciado	86 (8,0)	
Viúvo	217 (20,3)	
Ignorado/em branco	58 (5,4)	
**Escolaridade** (anos)		
0	188 (17,6)	<0,001
1-3	174 (16,2)	
4-7	302 (28,2)	
8-11	263 (24,6)	
>12	63 (5,8)	
Ignorado	81 (7,6)	
**Ocupação**		
Remunerada	562 (52,5)	<0,001
Não remunerada	467 (43,6)	
Em branco/ignorado	42 (3,9)	
**Macrorregião**		
Centro-Norte/Noroeste	577 (53,9)	<0,001
Sul	177 (16,5)	
Norte	154 (14,4)	
Oeste	86 (8,0)	
Leste	76 (7,1)	
Em branco/ignorado	1 (0,1)	

O número de óbitos predominou acima de 64 anos (352 óbitos/ 32,8%), seguido da faixa etária entre 40-49 anos (230 óbitos/ 21,5%) (p-valor<0,001); entre mulheres solteiras (346 óbitos/32,3%) (p-valor<0,001); com escolaridade entre 4 e 7 anos de estudo (302 óbitos/ 28,2%) (p-valor<0,001); na população parda com (673 óbitos/62,8%) (p-valor<0,001). Em relação à ocupação, 562 óbitos (52,5%) ocorreram em mulheres que eram remuneradas e 467 óbitos (43,6%) sem remuneração (p-valor<0,001). 

A maior mortalidade por macrorregião ocorreu no Centro-Norte/Noroeste (577 óbitos; 53,9%), seguida pela Sul (177 óbitos; 16,5%), Norte (154 óbitos; 14,4%); Oeste (86 óbitos; 8,0%), Leste (76 óbitos; 7,1%) (p-valor<0,001), sendo respectivamente os locais com mais óbitos: Cuiabá (26,2%), Rondonópolis (8,0%); Sinop (2,9%), Cáceres (4,0%); Barra do Garças (1,6%) (Figura 2).

**Figura 2:** Distribuição geoespacial dos óbitos por câncer de colo do útero, conforme o local de residência. Mato Grosso, 2013-2022

A maioria dos óbitos, 811 (75,7%), ocorreu em pacientes que receberam assistência médica (p-valor<0,001). Porém, apenas 394 óbitos (36,8%) tiveram investigação (p-valor 0,008). Observa-se que as mulheres tiveram acesso limitado à investigação ambulatorial, hospitalar e domiciliar (p-valor<0,001) (Tabela 3).

**Tabela 3:** Óbitos por câncer de colo do útero, de acordo com as variáveis: assistência médica, houve investigação, investigação ambulatorial, hospitalar e domiciliar. Mato Grosso, 2013-2022 (n=1.071)

Variáveis	n (%)	p-valor
**Assistência médica**		
Sim	811 (75,7)	<0,001
Não	24 (2,2)	
Ignorado/em branco	236 (22,1)	
**Investigação realizada**		
Sim	394 (36,8)	0,008
Não	312 (29,1)	
Ignorado/em branco	365 (34,1)	
**Investigação ambulatorial**		
Sim	49 (4,6)	<0,001
Não	1022 (95,4)	
**Investigação domiciliar**		
Sim	102 (9,5)	<0,001
Não	969 (90,5)	
**Investigação hospitalar**		
Sim	173 (16,2)	<0,001
Não	898 (83,8)	

## Discussão

O estudo analisou a tendência temporal e o perfil sociodemográfico da mortalidade por câncer de colo do útero no Mato Grosso entre 2013-2022. A tendência de variação na taxa de mortalidade foi estacionária. Houveram mais óbitos em mulheres acima de 64 anos, ensino fundamental incompleto, pardas, solteiras, remuneradas, residentes na macrorregião Centro-Norte/Noroeste, que receberam assistência médica e investigação.

No Brasil, o rastreio do câncer de colo uterino ainda é realizado pelo exame colpocitológico. Em 2023, o Brasil iniciou um “Projeto-Piloto de Controle e Eliminação do Câncer do Colo do Útero”, utilizando para triagem o teste HPV-DNA (15). O teste é considerado padrão-ouro pela Organização Mundial de Saúde desde 2014, por ser mais eficaz e sensível que a colpocitologia, ter menor custo, menos resultados falso-negativos e integrar as estratégias mundiais para eliminação da doença (16). Pode ser realizado a partir dos 30 anos, substituindo a colpocitologia, que fica reservada quando o teste HPV-DNA for positivo e, se negativo, repetido a cada 5 anos (17). 

Os benefícios na redução da incidência e mortalidade por câncer de colo uterino gerados pelo rastreamento com testes de detecção de HPV superam os danos causados pelo aumento de testes positivos e colposcopias. Eles permitem início de rastreamento em idade mais avançada com intervalos de testagem mais espaçados (18), além de poderem ser realizados por autocoleta. A autocoleta é de fácil realização, com menos desconforto e capaz de transpor barreiras geográficas (19).

Este estudo identificou tendência temporal estacionária, ao longo do período, na taxa de mortalidade no Mato Grosso entre 2013-2022. Entretanto, estudo de série temporal realizado no Brasil em 2023, demonstrou diminuição estatisticamente significativa nas taxas de mortalidade por câncer de colo uterino no país em 2020, em comparação com a década anterior, em mulheres acima de 40 anos (20). Essa estagnação pode indicar limitações nos programas de rastreamento e necessidade de estratégias mais eficazes para ampliar a adesão e acesso no estado.

Observaram-se poucos óbitos abaixo dos 25 anos, justificando a exclusão dessa população no rastreio. Acima de 64 anos, embora essa população não esteja contemplada no rastreamento, representa a faixa etária de maior destaque, indicando possível deficiência no rastreio na década anterior. Demorar no diagnóstico dessas mulheres pode aumentar a mortalidade. Estudo observacional analítico de 2023, realizado em São Paulo, analisou 207 resultados anormais de esfregaço cervical em mulheres com mais de 64 anos e observou que aquelas que não seguiram as diretrizes de rastreamento apresentaram maiores chances de desenvolver câncer e lesões precursoras, sugerindo revisão da faixa etária de rastreamento no Brasil (21). Esses achados ressaltam necessidade da busca ativa pelos agentes de saúde e campanhas para promover a realização da citologia em idade mais precoce, visando reduzir a mortalidade e custos do tratamento.

Estudo transversal no Paraná, em 2013, avaliou razões para mulheres não aderirem ao exame. Demora no atendimento, distância até a unidade de saúde, vergonha, constrangimento e desconforto no momento do exame foram os fatores relatados. Esses sentimentos podem ser decorrentes de constante repressão sexual, especialmente entre mulheres de faixa etária mais avançada, resultando na negligência de suas demandas ginecológicas e criando uma barreira ao conhecimento sobre a importância da prevenção (22). Realizar campanhas para desmistificar o exame, promover um ambiente acolhedor e capacitar profissionais para uma coleta humanizada com o mínimo de desconforto para a paciente são ações que poderiam contribuir para aumentar o engajamento do rastreamento.

Revisão de escopo realizada no Brasil, em 2022, analisou diferentes intervenções educativas para a prevenção do câncer de colo do útero e reforçou que discussões em grupo, palestras e folhetos informativos podem aumentar o conhecimento das mulheres e melhorar sua adesão ao rastreamento. Também mostrou aumento significativo nas taxas de rastreamento em intervenções lideradas por agentes de saúde (23). Focar em campanhas de conscientização para prevenção através da coleta de citologia e vacinação para o HPV e busca ativa de mulheres acima de 50 anos pelos agentes de saúde, pode ser uma estratégia para melhorar os índices de rastreamento no estado.

Destaca-se que mulheres acima de 40 anos não tiveram acesso à cobertura de vacinação contra o HPV, disponibilizada pelo Sistema Único de Saúde desde 2014 para meninas de 9-14 anos. Espera-se queda das taxas de mortalidade nos próximos anos, uma vez que o impacto da vacinação na redução da infecção pelo HPV tem se mostrado significativo mundialmente (24).

Dados do Ministério da Saúde de dezembro de 2024 demostram que o Brasil está aumentando a população imunizada com cobertura de 96,6% para meninas com 14 anos e 69,0% entre 9 e 14 anos (25). No estado, a cobertura vacinal foi considerada adequada (acima de 80,0%) apenas na primeira dose, com 58,8% na segunda dose, especialmente entre meninas de 16 anos (26). A vacinação em dose única, a partir de 2024, facilitará atingir a meta da cobertura vacinal do estado e maior adesão à vacinação (7), sobretudo entre os grupos que apresentaram maior queda na adesão à segunda dose.

No Censo do Instituto Brasileiro de Geografia e Estatística (2022), 58,0% da população mato-grossense se autodeclarara parda, justificando a maioria dos óbitos em mulheres pardas. Estudo ecológico observacional brasileiro de 2020, analisando a mortalidade por câncer de colo uterino entre 1996-2017, destacou a mortalidade na população parda nas regiões Norte e Nordeste e preta, no Sul e Sudeste. Na região Centro-Oeste, destacou-se a população indígena, divergindo do presente estudo. A cor da pele da população se relaciona ao risco aumentado de mortalidade, considerando os possíveis fatores socioeconômicos e culturais, a baixa cobertura de rastreamento, os tratamentos inadequados e o estágio avançado ao diagnóstico (27).

O estudo também destacou a maior prevalência de mortalidade entre mulheres solteiras. Possíveis hipóteses para esse dado é que o estado civil pode influenciar a qualidade de vida, saúde e taxa de sobrevivência. A menor mortalidade entre mulheres casadas está possivelmente relacionada ao maior apoio social e psicológico, além de geralmente adotarem hábitos de vida mais saudáveis (27). Também mulheres solteiras tendem a ter maior exposição à infecção do HPV devido à possibilidade de terem mais parceiros sexuais (20,28).

O maior número de óbitos ocorreu em mulheres com ensino fundamental incompleto. Dado semelhante foi encontrado em estudo descritivo retrospectivo realizado em Cascavel-Paraná, que analisou 90 óbitos, entre 2012-2021, dos quais 31,1% ocorreram na escolaridade de 4 a 7 anos (29). Esses dados são reforçados por uma revisão sistemática realizada no Brasil, em 2018, que analisou a relação entre escolaridade e câncer de colo uterino, destacando que as barreiras educacionais podem dificultar o entendimento das mulheres sobre a adesão, prevenção e processo de evolução da doença (30).

Dentre os óbitos que exerciam ocupações não remuneradas, observou-se maior proporção em donas de casa e, em menor número, estudantes e desempregadas. Estudo observacional em Recife-Pernambuco, em 2008, analisou 323 óbitos e demonstrou proporções ainda mais elevadas, com 69,0% dos óbitos entre donas de casa (31). Em consonância com os achados, outro estudo descritivo transversal, realizado no Nordeste, analisou 699 óbitos, entre 2013-2014 e constatou que mulheres que trabalham fora de casa têm participação maior no rastreamento (32).

Os resultados indicam baixa investigação e deficiência nos exames preventivos. Em Mato Grosso, 6,3% das mulheres entre 25-64 anos nunca realizaram o exame citopatológico. Na região Centro-Oeste, a realização foi de 78,8%, abaixo da média nacional de 81,3% (2). Porém, a maioria das mulheres que faleceram recebeu assistência médica, indicando a presença de uma rede assistencial oncológica no estado.

 Estudo descritivo no Brasil analisou a mortalidade por câncer de colo do útero entre 2012-2016, registrando 27.716 óbitos. As maiores taxas ocorreram no Norte e Centro-Oeste, onde a taxa caiu de 27,12 para 24,13 por 100 mil mulheres (33). Esses dados reforçam a alta mortalidade por câncer de colo uterino na região e indicam desafios na detecção precoce e acesso aos serviços de saúde.

A distribuição esparsa no Centro-Oeste dificulta o acesso aos serviços de saúde, exigindo estratégias para uma cobertura eficaz e equitativa (34,35). No Mato Grosso, um estudo descritivo mostrou que a cobertura da Estratégia Saúde da Família nos 141 municípios permaneceu abaixo de 70,0%. A Baixada Cuiabana, incluindo Cuiabá e Várzea Grande, registrou as menores taxas com aproximadamente 40,0%, evidenciando carência na rede primária (36).

Uma revisão sistemática de 2020 identificou barreiras ao rastreamento do câncer de colo do útero em áreas rurais, como distância, transporte e acesso a especialistas (37). O Mato Grosso, devido à sua extensão territorial, enfrenta desafios relacionados à distância entre os municípios menores e os principais centros de referência, problema que poderia ser mitigado com a utilização do autoteste para o HPV.

O alto índice de óbitos em alguns municípios pode ser atribuído à centralização dos centros médicos e serviços especializados em oncologia na capital do estado (36). Municípios menores podem enfrentar subnotificação ou falta de acesso a tratamentos adequados, levando à migração de pacientes para áreas com assistência médica (38). Este estudo mostrou maior número de óbitos na macrorregião Centro Norte/Noroeste, especialmente em Cuiabá. Um estudo observacional de 2023 confirmou essa tendência, destacando São Paulo com maior cobertura de rastreamento (20).

Como limitações deste estudo, a pesquisa foi realizada com dados secundários, podendo ocorrer viés de seleção por subnotificação dos óbitos ou ausência de diagnóstico. A notificação do câncer de colo uterino não é compulsória. A plataforma da Secretaria de Saúde de Mato Grosso só permite categorizar as variáveis de investigação como “sim” ou “não”, sem opções para detalhar ou excluir dados como “ignorado” ou “não informado”, podendo comprometer a precisão dos resultados. Além disso, o ponto de corte de 50% para redistribuir óbitos por neoplasia maligna do útero não especificada (CID-10, C55) é uma prática válida, difundida pela Organização Mundial de Saúde (13) e adotada pelo Instituto Nacional de Câncer (39), mas pode ser limitante. Ele assume uma distribuição igual entre câncer de colo e corpo do útero, podendo não refletir a realidade epidemiológica. Foram selecionadas apenas pacientes residentes no Mato Grosso, desconsiderando aquelas que possam ter mudado para tratamento em outros estados.

Para estimar o risco relativo de um evento como a morte, é necessário comparar dois grupos distintos: um que experimentou o evento (morte) e outro que não (sobreviventes). No entanto, os dados utilizados neste estudo foram extraídos de um sistema que registra apenas óbitos. Dessa forma, não há um grupo de comparação (sobreviventes), impossibilitando o cálculo do risco relativo de morte. Esse método requer a proporção de ocorrência do evento em dois grupos distintos para estimar a diferença no risco entre eles, não se aplicando ao presente estudo.

Este é o primeiro estudo que analisou a tendência temporal e o perfil sociodemográfico da mortalidade por câncer de colo do útero no Mato Grosso entre 2013-2022. Os resultados demonstraram uma tendência estacionária na mortalidade, apesar da tendência de queda observada no Brasil. Essa discrepância sugere que o rastreamento e políticas preventivas podem estar sendo menos efetivos para algumas populações. Como era esperado, características sociodemográficas, como faixa etária, raça/cor da pele e macrorregião de residência, descrevem o perfil dos óbitos, evidenciando desigualdades no acesso e na adesão aos programas de saúde.

Para superar esses desafios, sugerem-se estratégias como conscientização, ampliação da vacinação, introdução do teste molecular de HPV, capacitação profissional, busca ativa de mulheres acima de 50 anos para rastreamento. Tais medidas poderiam melhorar a detecção precoce, ampliar o rastreamento, reduzir a mortalidade e otimizar recursos. 

Espera-se que os resultados deste estudo apoiem o combate ao câncer de colo de útero no Mato Grosso. Compreender as características sociodemográficas dos óbitos pode orientar políticas de saúde direcionadas, reduzindo desigualdades regionais e promovendo uma prevenção mais eficaz. Estudos futuros devem avaliar adesão e impacto das atuais políticas de rastreamento, visando adaptar as intervenções às necessidades específicas do estado.

## Data Availability

O banco de dados e as variáveis de análise utilizados na pesquisa estão disponíveis em http://appweb3.saude.mt.gov.br/dw.
